# Considerations for the design of overdose education and naloxone distribution interventions: results of a multi-stakeholder workshop

**DOI:** 10.1186/s12889-023-15554-6

**Published:** 2023-05-15

**Authors:** Kate Sellen, Benjamin Markowitz, Janet A. Parsons, Pamela Leece, Curtis Handford, Nick Goso, Shaun Hopkins, Michelle Klaiman, Rita Shahin, Geoffrey Milos, Amy Wright, Mercy Charles, Laurie Morrison, Carol Strike, Aaron Orkin, SOONER Investigators

**Affiliations:** 1 Health Design Studio, OCAD University, Toronto, ON M5T 1W1 Canada; 2 Applied Health Research Centre, Li Ka Shing Knowledge Institute, St. Michael’s Hospital, Toronto, Canada; 3 Public Health Ontario, Toronto, Canada; 4 Department of Family and Community Medicine, University of Toronto, Toronto, Canada; 5 Inner City Health Associates, Toronto, Canada; 6 Toronto Public Health, Toronto, Canada; 7 Department of Emergency Medicine, St. Michael’s Hospital, Unity Health, Toronto, Canada; 8 St. Michael’s Hospital, Unity Health, Toronto, Canada; 9 Dalla Lana School of Public Health, University of Toronto, Toronto, Canada; 10 Department of Emergency Medicine, St. Joseph’s Health Centre, Unity Health, Toronto, Canada

**Keywords:** Opioid overdose, Overdose education, Harm reduction, Naloxone distribution, Stigma, Co-design

## Abstract

**Introduction:**

Opioid overdose epidemic is a public health crisis that is impacting communities around the world. Overdose education and naloxone distribution programs equip and train lay people to respond in the event of an overdose. We aimed to understand factors to consider for the design of naloxone distribution programs in point-of-care settings from the point of view of community stakeholders.

**Methods:**

We hosted a multi-stakeholder co-design workshop to elicit suggestions for a naloxone distribution program. We recruited people with lived experience of opioid overdose, community representatives, and other stakeholders from family practice, emergency medicine, addictions medicine, and public health to participate in a full-day facilitated co-design discussion wherein large and small group discussions were audio-recorded, transcribed and analysed using thematic approaches.

**Results:**

A total of twenty-four participants participated in the multi-stakeholder workshop from five stakeholder groups including geographic and setting diversity. Collaborative dialogue and shared storytelling revealed seven considerations for the design of naloxone distribution programs specific to training needs and the provision of naloxone, these are: recognizing overdose, how much naloxone, impact of stigma, legal risk of responding, position as conventional first aid, friends and family as responders, support to call 911.

**Conclusion:**

To create an naloxone distribution program in emergency departments, family practice and substance use treatment services, stigma is a central design consideration for training and naloxone kits. Design choices that reference the iconography, type, and form of materials associated with first aid have the potential to satisfy the need to de-stigmatize overdose response.

**Supplementary Information:**

The online version contains supplementary material available at 10.1186/s12889-023-15554-6.

## Introduction

More than 17,602 apparent opioid-related deaths occurred in Canada between January 2016 and June 2020 [1]. While opioid agonist treatment is widely available in Canada and has been shown to reduce opioid overdoses [2–6], the opioid crisis is escalating and has worsened during the COVID-19 pandemic with evidence showing that there have been further increases in fatal and non-fatal overdoses [7] during this time. Harm reduction services, crucial for the prevention of overdoses and other drug related harms, have also been interrupted during the pandemic [8, 9] leading to a dual crisis in overdose response programs.

Overdose education and naloxone distribution (OEND) reduces opioid-related deaths by equipping and training people who are likely to witness overdose to respond with effective first aid interventions, including responding by administering the opioid antagonist naloxone [3–5]. OEND programs are among a small set of interventions that have been shown to reduce opioid-associated mortality at the population level [2, 10]. While publicly-funded overdose education and naloxone distribution programs have been established in every province and territory in Canada [6, 11], there remain gaps in our knowledge about effective design and implementation of the components of these programs, including knowledge relating to awareness and access [12, 13], carrying and use [14] (including curricula needs [15]), regional and other variabilities [16]. While there are rapidly developing areas of research addressing these aspects of OEND programs [17], these have been described as currently underdeveloped [18].

Take-home naloxone and training is a key component of OEND programs and provision of take-home naloxone and related education has been offered as part of harm reduction programs and addiction services for some time [19]. Observational research of naloxone distribution and training programs suggests that lay people are highly capable of learning how to successfully administer naloxone in the community [20]. An observational study of overdoses in Massachusetts found a reduction in overdose mortality rates for communities that had implemented OEND programs compared to those that had not [21].

Within Canada, the reclassification of naloxone to a non-prescription medication made it easier for community-members to get access to naloxone through OEND programming [22–24]. Compared to the early 2000s, population wide studies in Canada reveal that naloxone has become more widely available across the country [25, 26] particularly through pharmacy OEND programs. While community pharmacies are key distribution points for take home naloxone in Canada and elsewhere [27, 28], OEND programs have expanded in emergency departments, harm reduction and community health centers and agencies. Despite these efforts, the Canadian opioid overdose epidemic is not yet slowing down [29] and has been exacerbated by the COVID-19 pandemic [6–8]. Implementing COVID-19 public health approaches to flatten the epidemiological curve of the pandemic led to disruptions in harm reduction services, particularly at sites for Naloxone distribution [30]. This has led to the consideration of additional venues or situations in which OEND programs can be implemented wherever at-risk patients receive care [5]. International guidelines call for naloxone distribution and overdose education among patients and communities at risk of, or likely to witness, opioid overdose but there is still a lack of supports available to implement these guidelines [31, 32].

### Objectives

The aim of this study is to explore how OEND programs could be better designed from a broad and multi-stakeholder perspective such that considerations can be identified for OEND supports. This study is part of the larger SOONER project Surviving Opioid Overdose with Naloxone Education and Resuscitation designing an OEND program for emergency department, walk-in and family practice clinics, and addiction medicine units.

## Materials and methods

Using a co-design/participatory approach to frame the process and structure of the study, we used a qualitative methodology of interpretive description to approach the research questions [33] listed below.

1. What are the characteristics and concerns of stakeholders in relation to opioid overdose?
2. What is the current experience of overdose in terms of awareness, access, and administration of naloxone?
3. How might stakeholders envision a better experience in terms of awareness, access, and administration of naloxone?

This inductive, constructivist approach was well-suited to the goals of the study as well as to the principles of collaborative co-design [34].

### Study design

The study activities took the form of both participatory design methods [35, 36] as well as more discrete co-design techniques [37, 38]. The study was centered around a workshop that included both organizational perspectives as well as the perspectives of those with lived experience of overdose and overdose response over 1 day of facilitated co-design activities. The questions guiding the structure of the workshop were deliberately open ended to elicit insights on general factors relevant to opioid overdose response and OEND programs. Participants in the workshops were supported using standard co-design materials (empathy maps, and experience maps) [39, 40] customized to reflect the questions above, and with facilitation by co-design specialists from [removed for review] [41, 42]. The workshop was structured into two sessions with participants organised into stakeholder groups (5 tables) for the first session working with empathy and experience maps [39, 40]. The second session (after eating lunch together) saw participants move to difference tables for multi-stakeholder discussion using the empathy maps and experience maps as supports for reflection and dialogue [38]. At least one person from each stakeholder group stayed with their original table as a representative of that stakeholder group and to help interpret the empathy maps and experience maps for other stakeholder groups.

### Setting

The workshop was hosted by the SOONER project at the Health Design Studio at OCAD University, with support from the Canadian Centre on Substance Use and Addiction. Ethics approval for the study was granted by both the relevant healthcare partners and academic partners including the Research Ethics Boards of OCAD University and St Michael’s Hospital (Unity Health).

### Sampling and recruitment

The participants were recruited from the pan-Canadian Working Group on Overdose for the SOONER project. People with lived experience of drug use and overdose were invited by the Canadian Centre on Substance Use and Addiction and nominated by SOONER community partners. Each table consisted of between three and five representatives, supported by three circulating research team members and a lead facilitator.

### Data collection

The workshops included access to and use of abstract materials (sticky notes, pens, stickers) and specific resources (prompt cards with questions supporting each of the three questions above and quotes from existing literature on OEND experiences). As with many co-design techniques workshops such as this one include general open-ended materials and tasks, and, more detailed materials and tasks that are designed for the specific needs of the study. These materials are intended to support dialogue and provide multiple opportunities for different participants to engage in the process of discussion [38, 40]. The visible results of the workshop (i.e., work done on paper) were photographed. During the co-design workshop, dialogue at each of five tables (five stakeholder perspectives) was audio-recorded. A professional transcriptionist transcribed each audio recording to capture conversations in a form as close to the original oral style as possible.

### Analysis

As researchers interested in the field of opioid overdose, we were aware of stigma experienced by people who use opioids [43]. As qualitative researchers, we employed stigma theory to guide study design and analysis [44] drawing on Link and Phelan’s work, recognizing stigma at the level of the individual, social relations, and structural/systemic levels [45]. We took an inductive, qualitative approach [46] to iterative analysis, forming and refining ‘themes’ [44]. Initially, two senior members of the research team ([removed for review]), reviewed and analyzed a subset of transcripts to identify initial codes and patterns, developing a preliminary coding framework [46]. A third qualitative analyst joined the team (([removed for review]) to apply the coding framework using NVivo [47] to manage teamwork and track discrepancies and questions. Regular meetings were held between the senior team members and the qualitative analyst to check for consistency in the application of the coding framework. Questions were tracked using memo notes, discrepancies were resolved by discussion, consensus building, and review. There was subsequent input from a fourth analyst (([removed for review]) on policy and public health practice in the realm of opioid overdose [48] to further support quality and rigour in the analysis process and to guide the next step (question development). We formed a list of questions through collective interrogation of the coded transcripts after the initial coding framework was applied (Table 1). These questions organized the coding framework in further abstractions and were used to refine core themes that further characterized the dialogue. Dye has described [49], this approach resembles a “kaleidoscope”. The goal of this analysis was to highlight key elements of knowledge sharing that took place amongst the participating stakeholders.

**Table 1:** Analytic questions framed according to what participants were discussing

Naloxone	Opioid Overdose
• Who should be distributing naloxone and training lay users? • What are barriers to distribution? • Why is naloxone not like other more mainstream medications? • How should naloxone be distributed? • How much naloxone is enough? • How is naloxone being administered? • When naloxone is used, what ensues?	• Who is at risk? • Who needs overdose response preparation? • What does an overdose look like? • What are barriers to responding? • Where do overdoses occur? • Why do people not intervene? • Why do people overdose? • Why is the overdose landscape changing? • How does the general public characterize opioid overdose? How do healthcare providers characterize it? • How is overdose care and education provided?

## Results

In total, 24 people participated in the workshop in stakeholder groupings including ‘family/friends of those at risk of overdose’, ‘emergency responders’, ‘people who take opioids’, ‘frontline/harm reduction or clinical and allied health practitioners’, and ‘program administration’. At points throughout the results, we delineate who is talking according to the following perspectives: persons who take opioids (PWTO), family/friends of PWTO, emergency medical services (EMS), law enforcement, providers (physicians, nurses, nurse practitioners and pharmacists), and harm reduction workers.

**Fig. 1:**
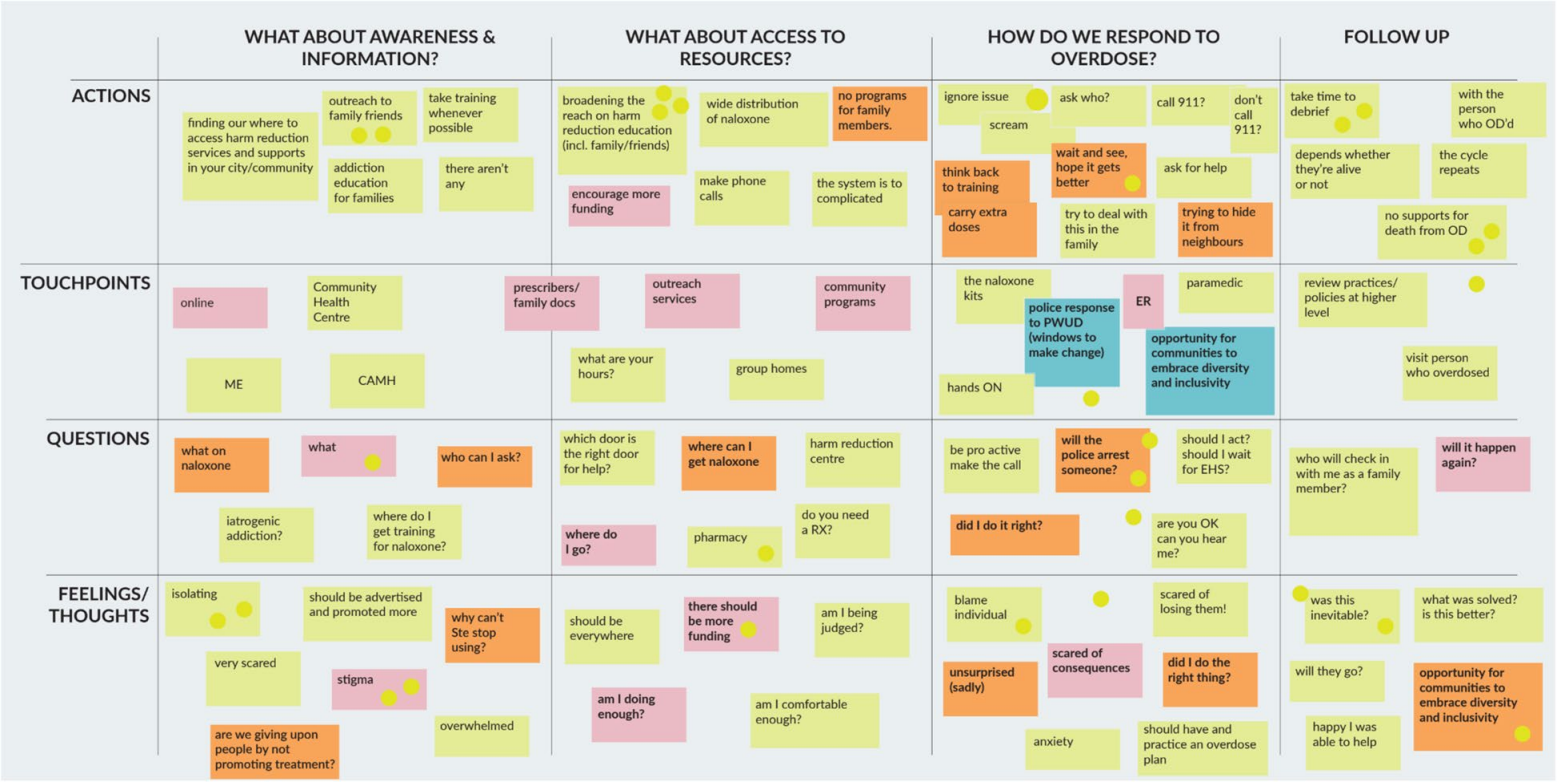
Journey map: family and friends of people with lived experience of overdose

The results of the analysis of the dialogue and participation indicated three themes covering naloxone administration and specific needs of response, training materials, and kits; issues of access, and issues of awareness, including stigma and public perception and support for naloxone programs. Each stakeholder group created an empathy map and journey map as part of the process to facilitate the workshop discussion, links to these artifacts have been included in as appendices/attachments to this paper. We provide an example below (Fig. 1).

The results indicate a variety of design considerations for OEND (Table 2 summarizes these considerations).

**Table 2:** Design Considerations and Requirements for OEND toolkit

**Considerations**	**Design requirements**
1. Recognizing an overdose may not be straight forward	• Training should support rapid response, • Include messaging that naloxone is safe to use in any unresponsive person, and will not cause any harm
2. Responders may not know “how much is enough”	• The kit should be designed so that the responder does not need to make dosing decisions
3. Stigma may reduce the likelihood and pace of response as well as the likelihood of asking for, giving out, and accepting the offer of a kit and training	• An anti-stigma approach in aesthetic choices, language use, and tone, is necessary to reduce potential barriers to response • The choice of nasal naloxone in take home naloxone distribution kits is suggested to reduce stigma, reduce potential training requirements, and increase likelihood of timely response
4. drug paraphernalia may be both stigmatizing and a potential legal risk to lay responders	• The choice of nasal naloxone in take home naloxone distribution kits is suggested to increase uptake among individuals who may be deterred by needles and ampoules
5. There is a need to move overdose training and response beyond the professional sphere and beyond those immediately at risk who may already be responding and comfortable with needle-based naloxone	• Design choices should position overdose response as a conventional first aid intervention,
6. There is high potential for overdose alone and there is a need to support the option of response by a friend or family member	• The design needs to support sharing with others (both training and kit), recognition as a first aid supply, positioning the kit as part of a safety plan
7. *Calling* 911 may be thought of as not a “safe” option for those that use drugs and their family and friends	• The training kit should emphasize calling 911, but also support response where 911 is not called

### Are community members (professional and lay-responders) prepared to respond?

Participants questioned whether community members (including police officers, service providers and lay people) are prepared to respond to opioid overdose. In particular, participants voiced concerns related to recognizing the physical signs of an overdose, knowing how much naloxone is enough, first-aid response, legal barriers, and overdose risk perceptions.

#### What does opioid overdose look like?

This had two aspects that participants emphasized – one had to do with *recognizing the signs of overdose* and the other had to do with *stigma* associated with the circumstances in which overdose occurs. Participants expressed concerns that lay people along with service professionals (police officers or security guards in particular) may experience difficulties recognizing an overdose. A harm reduction worker noted that ‘mixed’ or ‘atypical presentations can challenge responders’ preconceived notions of what overdose looks like*.* Similarly, discussions at the family/friends table, corroborated that opioid overdoses can be difficult to recognize:

*Family/friend of PWTO: …some of the overdoses have an odd presentation as well, because now we're getting the fentanyl and carfentanil [mixed] in the crystal meth,…*

Family and friends of PWTO worried that stigma and contextual factors may interfere with recognizing and responding to an overdose, potentially amplifying reluctance to respond. They portrayed overdose as disordered and used stigmatising terms to describe it, describing the potential for a “messy or dirty scene” with many distractions (e.g., the smell of alcohol, the presence of needles, and the sight of vomit), which could deter first-responders from acting, or acting in a timely way. They described this as manifesting in a reluctance to touch someone in overdose if drug paraphernalia are present, for example:

*Family/friend of PWTO: If there's education around what an overdose looks like, that's a good thing, but it still contributes to the stigma. Because, if somebody has vomit around them, nobody's going to touch them. If somebody has syringes on them, nobody's going to touch them.*

#### How much naloxone is enough?

With respect to administering naloxone, participants raised questions as to how much naloxone is enough to counter the potent effects of synthetic opioids like carfentanil. A harm reduction worker recounted:

*When you're talking about the .. synthetic opioids, like, we're now carrying six vials with us. And, even with that, we had an incident a couple of weeks ago where the nurse I was with went through all six.*

Moreover, the form in which naloxone is administered may influence one’s perception of dose response. A PWTO voiced skepticism over nasal naloxone, and if it was as effective as injection,

*…when you overdose, your breathing [is] shallow, you know what I mean. So, if you can't take a breath in, is the spray going to do anything, right?*

Ongoing work needs to be done to provide opportunities to address questions about naloxone use and how it works. Opposite to these concerns about not giving enough naloxone, participants also discussed the notion of withdrawal associated with too much naloxone. For example:

*Family/friend of PWTO: …once people are overdosed, when they come to after the naloxone, they're very agitated. They have pain, they're angry and they get instant withdrawal. So, learning how to deal with somebody after they come out of an overdose and what we can do …*

Participant accounts related to this issue of withdrawal suggest that administering naloxone may be perceived as a balancing act. A harm reduction worker recounted their approach to this issue:

*… use one dose and see what happens. Don't panic and give all three doses because otherwise you end up putting somebody into withdrawal and they're not happy. So, it's almost like titrating. Give one dose, carry on with your resuscitation and then give another dose.*

#### Resuscitation and feeling prepared

Aside from administering naloxone, participants spoke about a broader first-aid response. PWTO and their family/friends wanted to empower themselves with the necessary first-aid knowledge and skills to effectively respond in an overdose situation. This involved learning how to take command:

*Family/friend of PWTO: So, if you're there and you're the quarterback, you can, kind of, like do the playback for that. 'Hi, I'm [name]… I have experience with this, I'm going to take control of the situation right now. Would you please go [call] 911 and call paramedics for us? …*

Similar to having a fire safety plan in one’s home, family and friends spoke about a desire to implement opioid overdose safety plans as common practice within communities:

*Family/friend: if you have a Fire plan for your house, why not have an overdose plan? You're in charge of calling. You've got to get the kit. You're staying with the person. Something like that?*

To help guide lay responders to prepare for resuscitation, healthcare providers spoke about developing their own overdose response instructions to accompany the naloxone kits they were distributing:

*Provider: This is something we made up for our kits'… So, you do, 'shake and shout'…if you don’t get a response, then you call 911. Then, you administer naloxone. Then, you check breathing. If they're not breathing, then you do chest compressions for two minutes, then administer a second dose.*

Although healthcare providers spoke about educating lay people, they expressed for themselves uncertainties over resuscitation. An EMS participant noted,

*EMS participant: … we're just sort of questioning what is the best sort of chain of survival for this person? And, it's beyond just that, but is it call 911 first? Is [it] deliver Naloxone first? Is it start ventilations? If that's the case then we need to start properly training people…*

In addition, prior to the moment when an individual enters into overdose, a family/friend of PWTO indicated that it was also critical to prepare a “using plan” as a preventive component of a plan,

*Family/friend: I was just talking to the Chief [Indigenous community]… and he told me, the people that died of the carfentanil recently… were from his reserve…And he said, "I don't know why they didn't have someone try it out first instead of all of them doing it." … he said they should have a plan before they're going to use if they don’t know who they're buying those drugs from, right? …*

Rather than contextualize overdose education and naloxone distribution programs solely around the acute episode of overdose (i.e. when a person requires resuscitation), this quote suggests that public health initiatives could benefit from widening their scope to prevention and preparedness. This seems especially valuable as participants highlighted barriers to calling 911.

#### Barriers to calling 911

Participants indicated that calling 911 is an integral part of an overdose response within a community setting. Layperson accounts highlighted a lack of trust between police officers and some community members, as people might fear that police officers could press drug-related charges against them at an overdose scene, even for carrying naloxone. As one PWTO recounted:

*I almost got arrested because of my naloxone kit. None of them [police] knew what it was. None of them knew anything about it… we have people that, because of the stigma of law enforcement, they administer Narcan, don't tell anybody and leave.*

Feeling a need to flee the scene after calling 911 and perceiving themselves to be at legal risk when providing a potentially life-saving intervention (despite the Good Samaritan Act [50]) makes this aspect of designing overdose education interventions particularly difficult. For lay participants who spoke about calling 911, they shared their own approaches regarding how to communicate an overdose situation,

*Family/friend of PWTO: …do not say that they overdosed. Just say, 'my friend is not breathing.' Because, chances are, the ambulance may not call the cops unless they see, you know, like, a horrific scene, right? .”'*

Upon hearing these accounts related to fear of police accompanying EMS to overdose scenes, a representative of law enforcement explained why police were needed at the scene.

*Law enforcement: It has nothing to do with the criminal aspect. It's for provider safety, right. So, that to me is very powerful, you know, people that aren't saying it's an overdose just because they think there's gonna be a legal ramification and that's putting responders into an unsafe position.*

Although members of law enforcement may see police presence as helpful in ensuring safety for first responders, other participants spoke about their presence as intimidating. To combat fears concerning law enforcement, a healthcare provider spoke about adapting their educational approach to account for this context, and how this was an additional burden for providers and harm reduction workers.

*Provider:...if you call in and you're in an apartment with a lot of drug paraphernalia, you drag that patient out…the person out into the hallway, you lock your door and you do your first aid there until EMS arrives, and then you leave. ….sort of like a temporary measure until law enforcement really puts in the effort to build a trusting relationship with the community ..”*

Participants in this study indicated that legal concerns and stigma need accounting for when thinking about how to implement overdose response training into communities, more so than in other situations that would require calling emergency services.

#### Overdose risk perception

Although participants across tables generally implied that there was a “typical” individual at risk for overdose (i.e. people on high-dose opioid prescriptions or who inject drugs), participants emphasized that naloxone administration and overdose response should be viewed as a broader community health concern—one that could impact anybody using drugs or taking prescription opioids. For instance, there was talk amongst law enforcement participants about overdoses that affect opioid-naïve individuals, such as those who are using illicit recreational drugs tainted with synthetic opioids.

*EMS/law enforcement: So, for an example, in British Columbia, everybody and their dog focuses on the Downtown Eastside, but in reality, these issues are anywhere there's a drug element, right? And, that started with cocaine.*

Moreover, a provider proclaimed that the general public should widen their scope when thinking about who needs naloxone,

*…there's no one population. It's across the spectrum of gender. It's across the spectrum of income levels. It's across race, ethnicity. So, it's not just low-income individuals who are mostly suffering from overdose. But, you have, like, Bay Street professionals who have just as much risk.*

Although providers felt able to identify patients at risk for overdose, they felt that convincing individual patients of this risk was a difficult endeavor,

*Provider: I have a lot of patients who will have high-dose prescribing and benzodiazepine and, in my view, be at really high risk because of what we know from research. But, they don't feel like they're at risk because they've never overdosed before because their doctor is prescribing it. ..'*

Broadening the scope of who is actually at risk of overdose and recognizing the opioid crisis for the public health emergency that it is seems vital to designing an effective educational intervention that does not feed into stigmatizing views or attitudes surrounding those who overdose.

### Moving naloxone out of the professional sphere

Within discussions, participants conveyed a desire to make naloxone a community-based resource; Participants expressed that naloxone should be readily accessible to a broad range of people. As one harm reduction worker explained,

*It should be the people for the people. We shouldn't be delineating, you know, pharmacists are the one[s] that should be delivering this. It should be people for people and..*

Unlike other first aid interventions such as public access to automatic external defibrillators (AED), participants suggested naloxone was not yet normalized within communities. For instance, a PWTO said,

*…you can't get naloxone unless you say you're an addict. Or, supporting an addict and then the addict has to come with you. Like I think that it should be available to whoever wants it.*

In addition to participants with lived experience, providers likewise expressed concern that naloxone was not easily available in public spaces where overdoses have occurred,

*Provider: Well, I mean you could even go as far as having it like an AED, right? We have it [AED] all in community centres. Why don't we have naloxone boxes that you could put beside your AED, with training? Or parks, or anywhere. Or, downtown Toronto on Yonge Street, you know? Anywhere.*

Participants discussed how accessing naloxone could be difficult for individuals due to the centralized nature of distribution via pharmacies. Providers discussed the alternative approach of peer distribution,

*Provider: Another issue that we've seen ..,, was being able to train peers or people who are users and live in buildings where there's high use, to be able to teach them, like, have the certification, the requirements, the authorization if you will, like, from a particular organization to distribute naloxone. ... So, I think peer distribution of naloxone is huge.*

Furthermore, jurisdiction could ultimately dictate how readily available (and accessible) naloxone is. Taking Ontario as an example, a provider indicated that there are northern regions of the province with relatively few pharmacies per capita, yet these communities have a disproportionately high concentration of individuals at risk for overdose,

*Provider: … we are hit really hard by opiate addiction. So, there has to be a way of rolling out naloxone. And, we use what we call 'unregulated care providers' to deliver our Suboxone programs…So, we actually train what we would call 'lay people' to be able to do medical things….*

Participants emphasized that community-based (or peer) distribution of naloxone should be an essential component of intervention design, as it would help naloxone move from the professional sphere (such as pharmacies) and allow it to become a more readily accessible first-aid medication.

### Narrative around naloxone

In discussions, what seemed to hinder the wide-spread uptake of naloxone within community settings was stigma, whereby naloxone is commonly associated with injection drug use, and PWTO are prone to encounter negative perceptions from others. For example, a person with lived experience believed that naloxone would only start to become more widely accepted if it played to the hearts of Canadians and was seen as a life-saving medication that could be used to save someone you love,

*…when that young woman… a couple years ago died, then everybody was talking about Naloxone, right? And, that's when you hear about naloxone…*

This “young woman” portrayed above is described as an unlikely victim of overdose. The participant presents them as someone who the public would not normally expect as needing naloxone. Contrary to this example, a provider chose to highlight what appear to be ‘repeat offenders’ who health care providers can lose compassion for,

*Provider: We definitely see the stigma associated with it even within our own practice. ‘This is not our job.’ ‘We don't have time to deal with this.’ ‘This is your 'get out of jail free card.' ‘This promotes drug use’. We hear this and we see this in our practice. So, there's blaming and shaming with individuals who use [and use naloxone].*

Furthermore, providers spoke about difficulties generating public support for naloxone and harm reduction programming,

*Provider: When you think of all the funding and public service announcements that go into car safety; seatbelts, roads, traffic, all the money that goes into keeping us safe on the road. ...But, we can't accept that people are going to continue using [opioids]. They're not just going to stop, so how do we figure that out? It's a massive problem.*

The core goal of using naloxone is to save a person’s life, yet participants indicated that an abstinence narrative had detracted attention away from accomplishing this fundamental goal:

*Person with lived experience: ... it's that fear of, if I'm using by myself and I OD what do I do, you know? That's a big fear. So, yeah. Especially when you've been clean and you know your family and friends know that you've been clean, you don't want to phone them up and say ‘I'm going to use, can you call me in half an hour and make sure I'm okay?’ ...*

Here we see that PWTO do not always feel comfortable opening up to others (especially family members) about their drug use practices, and subsequently, use alone. Alternatively, some PWTO may take a more advocative stance against an abstinence narrative making naloxone more readily available,

*Person with lived experience: She [girlfriend] was the best advocate, you know. She was such an advocate for it, you know like every time that we used together, we'd always say , ‘hey I've got a kit’ and she'd be like, ‘yep I've got one too.’*

This participant perceived social relationships and interactions as critical in terms of the uptake of naloxone, underscoring the value of supporting social relationships and ties for OEND programs.

## Discussion

The results from the work described in this paper led to insights informing the design of an integrated solution for an OEND program combining elements across digital and physical media for use in family practice, emergency departments, addictions clinics and community settings [41, 42, 51]. The multi-stakeholder perspectives on OEND detailed here reveal important considerations for designing an educational intervention tailored to lay bystanders responding to opioid overdose.

One of the key merits of our study is that it brought together a wide range of relevant stakeholders to share their expertise, experiences and perspectives. Workshop attendees spoke to community members’ preparedness to respond to opioid overdose in terms of: lay responders’ capacity to recognize an overdose; naloxone distribution was characterized as only one part of a more complex resuscitation response, which included preventative strategies; perceived legal ramifications and risks associated with calling 911; as well as the role stigma plays in responders’ willingness to intervene.

Participants commented on access to naloxone as a major concern, whereby some saw an over-reliance on pharmacies for dispensing as problematic, which confirms existing research [25, 26]. The issue of access is further compounded in more remote communities where pharmacies themselves can be scarce. Several barriers preventing communities from having equal access to naloxone and overdose education identified in existing literature [2, 4, 5] were confirmed by participants. These barriers reflect the need for a holistic approach to OEND programming that addresses the complexity of stigma and how it is experienced on an individual level by understanding the larger social, political and legal system surrounding drug use [27, 28]. Participants recounted how the stigma surrounding opioid use extended to naloxone as an intervention, differentiating it from how (for example) AED or Epi-Pens are perceived. Abstinence discourses [52] were seen as a barrier to naloxone uptake, in that carrying naloxone implied illicit opioid use. Participants noted that opioid addiction and the need for naloxone extends across all sectors of society.

Stigma theory [53, 54] informed our analytic approach to this dataset. Recognizing the role stigma plays as it relates to the ongoing opioid crisis is of critical importance when it comes to intervention design. As Link and Phelan [54] noted almost 20 years ago, “stigmatization probably has a dramatic bearing on the distribution of life chances in such areas as earnings, housing, criminal involvement, health and even life itself.” This study reinforces existing work on stigma rather than uncovering new ideas about stigma. We chose to focus on how the discussions on stigma illuminated key features to consider in relation to designing an OEND intervention. Unfortunately, a predominant narrative [55] surrounding PWTO as ‘addicts’ persists in Canadian society [48]. This workshop highlighted that the pervasive stigma surrounding opioid use and opioid overdose remains a significant barrier to the uptake of both naloxone kits and overdose resuscitation efforts. A clear message emerged from the workshop: the integration of an anti-stigma approach is necessary in designing interventions aimed at addressing the opioid crisis.

Addressing the issue of broadening naloxone awareness and access, participants discussed how patients taking opioids may believe that there is a certain ‘type’ of person at risk for overdose, and that because they do not identify as a person ‘at risk’ they may be reluctant to accept naloxone. In combination, this implies attention needs to be paid to specific design choices that acknowledge and resist attributes that indicate either marginalisation or mainstreaming. This would suggest positioning a design as an opportunity for shifting the narrative on overdose, whilst being supportive of a harm reduction approach. Finally, broadening the scope of who is actually at risk of overdose and recognizing the opioid crisis for the public health emergency that it is vital to designing an effective educational intervention.

OEND programs were launched across the country at a rate that has made it difficult to evaluate individual quality and implementation, and compare effectiveness, making an assessment of equitable naloxone distribution infeasible [11]. Very little work exists to help guide the design of didactic materials to support OEND, the exception being a recent study from the Food and Drug Administration (FDA) in the USA on product labelling to educate lay people on steps to follow for effective administration of naloxone [28] and more recent work on needle-based naloxone instructions [56]. OEND initiatives such as that described by the FDA are meant to strengthen lay person capacity to successfully administer naloxone. Before an individual even sees a label, it is vital that programs exist to get naloxone into the hands of those who are in situations to use it.

This work highlights the need to address stigma as central to the design process and outcome of an OEND intervention that to support overdose response among a broader public. Design choices must broadly move naloxone, overdose, and overdose first aid beyond aesthetics and language. Design choices that reference the iconography, type, and form of materials associated with first aid have the potential to satisfy the need to de-stigmatize overdose response [51, 56]. To design effectively in a context of specific tensions and stigma, a co-design approach is appropriate, including the continuous engagement of both community members and staff in the point of care settings where OEND is proposed [51]. Moving beyond the design process and evaluation of the OEND kit and training, the results suggest a high need for translation of the work of this project through public engagement and knowledge mobilization activities. 

### Limitations

It is important to note that the context was urban Toronto in terms of lived/living experience representatives, while paramedics, program directors, police officers, allied health were not exclusively working in Toronto. There was significant representation from Vancouver whose experience of the overdose crisis was more longstanding but which may differ from other jurisdictions who wish to use the considerations for their OEND programs due to availability of naloxone and other factors. We were fortunate to have participants who were able to bring perspectives from other jurisdictions, remote communities, Indigenous communities, western Canada, recognizing that the local urban context dominated the discussion which may impact the applicability of the considerations to other contexts. Naloxone suppliers were not represented at the workshop.

## Conclusion

OEND kits emerged to support communities at risk of opioid overdose, and in the context of specific tensions and stigma. OEND programs created for overdose response that also consider stigma might be accomplished in part through the deliberate redesign and co-design of interventions such as training and naloxone distribution programs used to address the crisis –- an effort to effectively "design away" stigma. In this study we have been able to elucidate seven design considerations with actionable design requirements for detailed kit and training design including those that address stigma more directly. While stigma was not the primary focus of the research, it is a pervasive factor in naloxone awareness, access and overdose response.

## Supplementary Information

**Additional file 1.**

## Data Availability

The data gathered from the data gathering process and workshop will not be made publicly available. This is to ensure the privacy and confidentiality of our study participants is protected. However, datasets used and/or analysed during the current study are available from the corresponding author on reasonable request.

## Abbreviations

OEND: Opioid overdose education and naloxone distribution
COVID-19: Coronavirus disease 2019
PWTO: Person who take opioids
EMS: Emergency medical services
OD: Overdose
OD’d: Overdosed
AED: Automated external defibrillator
FDA: Food and Drug Administration
