# Supplementary material for: Considerations for the design of overdose education and naloxone distribution interventions: results of a multi-stakeholder workshop
Source: BMC Public Health. 2023 May 15;23:888. doi: 10.1186/s12889-023-15554-6 (PMC10183687; doi:10.1186/s12889-023-15554-6)
Supplement: Supplementary file 1 — Additional file 1. [file 12889_2023_15554_MOESM1_ESM.docx]

# Consortium Membership – SOONER Investigators:

# Kate Sellen,^1^ Nick Goso^1^, Mercy Charles^2^, Laurie Morrison^2^, Benjamin Markowitz^2^, Janet A Parsons^2^, Kristine Norris^2^, Rekha Thomas^2^, Kevin Thorpe^2^, Peter Jüni^2^, Pamela Leece^3^, Suzanne Turner^4^, Curtis Handford^4,9^, Shaun, Hopkins^5^, Rita Shahin^5^, Michelle Klaiman^6^, Geoffrey Milos^7^, Carol Strike^8^, Aaron Orkin^7,8,9,10^, Leigh Chapman^11^, Amy Wright^12^, Douglas M Campbell^13^, Vicky Stergiopoulos^14^.

# Affiliations:

Health Design Studio, OCAD University, Toronto, Canada^1^, Applied Health Research Centre, Li Ka Shing Knowledge Institute, St. Michael's Hospital, Toronto, Canada^2^, Public Health Ontario, Toronto, Canada^3^, Department of Family and Community Medicine, University of Toronto, Toronto, Canada^4^, Toronto Public Health, Toronto, Canada^5^, Department of Emergency Medicine, St. Michael's Hospital, Unity Health, Toronto, Canada^6^, SOONER Advisory Committee, St. Michael's Hospital, Unity Health, Toronto, Canada^7^, Dalla Lana School of Public Health, University of Toronto, Toronto, Canada^8^, Inner City Health Associates, Toronto, Canada^9^, Department of Emergency Medicine, St. Joseph's Health Centre, Unity Health, Toronto, Canada^10^, Population Health & Social Medicine Program, University Health Network, Toronto, Canada^11^, Toronto Metropolitan University, Toronto, Canada^12^, Allan Waters Family Simulation Centre, Unity Health Toronto, Toronto, Canada^13^, Centre for Addiction and Mental Health, Toronto, Canada^14^.
